# Impact of “dry sowing and wet emergence” water regulation on cotton soil water and salt dynamics, root growth and yield

**DOI:** 10.3389/fpls.2025.1685785

**Published:** 2025-10-24

**Authors:** Qiang Hu, Yu Xiao, Huqiang Li, Xiaofeng Wang, Jiao Lin, Wenqing Zhao, Zhiguo Zhou, Guodong Chen, Lu Han, Nan Cao, Sumei Wan

**Affiliations:** ^1^ College of Agriculture, Tarim University, Alar, China; ^2^ Key Laboratory of Genetic Improvement and Efficient Production for Specialty Crops in Arid Southern Xinjiang of Xinjiang Corps, Tarim University, Alar, China; ^3^ College of Agriculture, Nanjing Agricultural University, Nanjing, China

**Keywords:** emergence water management, soil water and salt dynamics, root vitality, antioxidant enzyme activity, yield

## Abstract

**Background:**

Xinjiang, with unique favorable conditions for cotton growth, faces challenges like water scarcity and soil salinization. The dry sowing and wet emergence (DSWE) water regulation technology may alleviate regional water shortages, but its impacts on soil water-salt dynamics, soil desalination rate (SDR), cotton root growth, yield, and irrigation water production efficiency (IWPE) in saline-alkali land remain poorly documented.

**Methods:**

A two-year field trial was conducted during 2023 and 2024, involving three different seedling irrigation amounts (W1, 22.5 mm, W2, 37.5 mm, and W3, 45.0 mm) and two drip irrigation frequencies (F1: one-time irrigation and F2: two-time irrigation), resulting in a total of six irrigation combinations, and a local spring irrigation amount was conducted as CK.

**Results:**

Results demonstrated that under the same emergence water amount, F2 treatment exhibited higher soil moisture content and lower soil salt content compared to F1. Increased irrigation frequency and enhanced emergence water amount (W2F2 and W3F2) had greater root length density (RLD) and root vitality, but a lower root-shoot ratio. The W2F1 and W3F1 treatments significantly increased the activities of peroxidase (POD), superoxide dismutase (SOD), and the content of malondialdehyde (MDA) in cotton roots. In contrast, the activities of POD, SOD, and catalase (CAT), as well as the MDA content in cotton roots under the W2F2 and W3F2 treatments were comparable to those in the CK, indicating no obvious physiological stress. Compared with F1, F2 significantly increased cotton boll number and seedcotton yield by 23.3% and 23.5%, respectively. Notably, however, there were no significant differences in boll number and seedcotton yield among the CK, W2F2, and W3F2 treatments, suggesting that increasing water amount did not further improve yield.

**Conclusion:**

In conclusion, DSWE technology maintains optimal soil moisture levels, thereby improving root system development, while simultaneously leaching salts from the rhizosphere and reducing oxidative stress. Under the current experimental conditions, the W2F2 treatment emerges as the most effective strategy for regulating seedling emergence water, effectively integrating water conservation, salinity reduction, and seedling vigor enhancement.

## 1 Introduction

The shortage of water resources has become a global issue, which seriously hinders the agricultural development in many arid and semi-arid regions (Rosa et al., 2020). Xinjiang, one of the main cotton-producing regions in China (Kang et al., 2023), and is a typical arid area characterized by low precipitation and scarce freshwater resources (Li et al., 2022). These factors lead to serious secondary salinization of cotton field soil, which restricts the sustainable development of cotton production in Xinjiang (Wang et al., 2024). Irrigation serves as a core regulatory measure in cotton cultivation techniques (Zuo et al., 2023). In current agricultural practices, winter-spring irrigation constitutes about 50% of total water use in southern Xinjiang. This irrigation strategy has a certain effect in alleviating soil salinity. However, excessive water use often causes the groundwater level to rise, thereby triggering more severe salinization problems (Yang et al., 2016).

To address the challenges of water scarcity and soil salinization, the dry sowing and wet emergence (DSWE) technology has been developed. The DSWE technology transforms the original winter-spring irrigation model for water storage, soil moisture preservation, and salt-alkali washing into a mode of film mulching for moisture retention and drip irrigation for moisture supplementation during sowing (Ma et al., 2024). It has remarkable advantages in water resource conservation, improvement of cotton seedling emergence rate, and promotion of yield and income increase (Wang et al, 2024). Ma et al. (2024) reported that under the DSWE mode, the high-frequency drip irrigation treatment (4 times) significantly increased cotton plant height, dry matter accumulation, and yield by 8.7%, 16.4%, and 15.9%, respectively, compared with the low-frequency treatment (2 times). Meanwhile, the high water amount treatment (15 mm) at the seedling stage could improve cotton yield by 14.3% compared with the low water amount treatment (6 mm). Compared with the low-frequency irrigation treatment, the high-frequency irrigation treatment can significantly improve the net photosynthetic rate and stomatal conductance of cotton leaves, with increases of 12.2% and 6.7%, respectively. Compared with traditional winter irrigation, fewer and more frequent irrigations treatment (with 15 mm of emergence water and a drip irrigation frequency of 4 times) resulted in only a 2.0% and 3.0% decrease in the average lint cotton yield and seedcotton yield, respectively, over two years, while the irrigation water consumption during the growing period was reduced by 38.5% (Ding et al., 2024). This advantage is closely related to soil moisture regulation. In the DSWE mode, the synergistic effect of high-frequency irrigation and high emergence water application can significantly increase the soil moisture content in the 0–30 cm shallow layer. Specifically, compared with the traditional winter irrigation treatment, fewer and more frequent irrigations treatment increased soil moisture by 10.2%. In terms of root growth, the increase in emergence water amount and irrigation frequency has a positive impact on root morphological development, the root length density (RLD) of the fewer and more frequent irrigations treatment was 18.7% higher than that of the traditional winter irrigation treatment, with a more extensive root distribution range (Ding et al., 2024). Furthermore, the drip irrigation frequency and emergence water amount in DSWE technology are positively correlated with soil moisture and RLD, which in turn improves cotton emergence rate and yield (Ma et al., 2025).

Based on the characteristic that salt moves with water (Hou et al., 2022a), soil moisture acts as a carrier for the migration of soil salts (Wei et al., 2025). Therefore, the distribution of soil salts is susceptible to the influence of irrigation regimes and follows the pattern of soil water flow (Wang et al., 2018). Xiao et al. (2016) found that drip irrigation with a lower soil moisture threshold had a significant positive impact on soil salt regulation. Cotton roots are the main organs for water uptake (Luo et al., 2024), and the migration paths and distribution patterns of soil water and salt directly affect their distribution characteristics and physiological activities (Ma et al., 2025). As an important underground organ, cotton roots play a core role in growth, development, and yield formation. Through the composite functions of absorption-transportation-regulation-support, roots serve as a key link connecting the soil environment and aboveground growth (Chen et al., 2018). Numerous studies have demonstrated that drought and salt content have a significant impact on the antioxidant enzyme activity of root systems (Noreen and Ashraf, 2009; Dai et al., 2020; Farhangi-Abriz and Torabian, 2017). However, research on the relationship between root vitality, antioxidant enzyme activity and the distribution of soil water and salt in saline-alkali land under the DSWE mode remains relatively scarce.

Most existing studies have explored that cotton growth, yield, quality, and root distribution under the DSWE technology. However, research on the effects of DSWE technology on cotton root growth, soil water and salt distribution, soil desalination rate (SDR), and irrigation water production efficiency (IWPE) in saline-alkali land remains scarce. Facing the increasingly severe problem of water resource scarcity and soil salinization, it is essential to develop and promote efficient water-saving and salt-reducing technologies, thereby providing a theoretical basis and technical support for an irrigation system characterized by the coordination of water-fertilizer-root-canopy. The aims of the this study were to: (1) investigate the effects of different emergence water treatments on soil water-salt dynamics, SDR, seedling emergence rate, cotton root growth, yield, and IWPE; (2) identify the optimal emergence water regulation scheme for the new water-saving, salt-reducing, and seedling-strengthening irrigation technology of DSWE under current conditions.

## 2 Materials and methods

### 2.1 Experimental site and design

The field study was conducted in Southern Xinjiang Industry-Education Integration Modern Agriculture Training Base of Tarim University, Alar (44°32′N, 81°18′E), China, from 2023 to 2024. This study region features a warm temperate, highly continental arid desert climate. It is marked by an annual evaporation rate ranging from 1,976.6 to 2,558.9 mm, while annual precipitation falls within the range of 40.1 to 82.5 mm. The 0–20 cm topsoil layer was characterized as sandy loam with a pH of 7.9, soil electrical conductivity (EC) of 3.9 dS m^−1^, organic matter of 13.2 g kg^−1^, available phosphorus of 14.2 mg kg^−1^, available potassium of 154 mg kg^−1^, and available N of 174 mg kg^−1^. The soil physical properties of the experimental plots are detailed in **Table 1**.

**Table 1:** **S**oil physical and chemical properties before irrigation.

Soil depth (cm)	Soil texture			Bulk density (g m^−3^)	Soil volume moisture content (%)	Field water holding capacity (cm^3^ cm^−3^)
	Clay	Sand	Silt			
0-20	2.73	56.03	41.24	1.59	13.39	0.23
20-40	2.68	56.01	41.31	1.63	16.20	0.21
40-60	2.89	54.21	42.90	1.58	16.95	0.24

The experiment deployed a completely randomized design with six irrigation combinations treatments and a local spring irrigation treatment as a CK (**Table 2**). Six irrigation combinations treatments included three different seedling irrigation amounts (W1, 22.5 mm, W2, 37.5 mm, and W3, 45.0 mm) and two drip irrigation frequencies (F1: one-time irrigation and F2: two-time irrigation). For F1, drip irrigation was applied once at the emergence stage, while F2 was drip irrigation twice at the emergence stage and the strong seedling stage (**Table 2**). For CK treatment, with an irrigation amount of 225 mm base on local spring irrigation amount was conducted in March of each experimental year. Each plot area was 69 m^2^ (4.6 m × 15 m) and three repetitions. Cotton (cultivar).

**Table 2:** Experimental design of the dry sowing and wet emergence for 2023-2024.

Treatment	Irrigation Quota (mm)			Seedling Water quantity (mm)
	Spring irrigation	Emergence stage	Strong Seedling stage	
W1F1	0	22.5	0	22.5
W2F1	0	37.5	0	37.5
W3F1	0	45	0	45.0
W2F2	0	22.5	15	37.5
W3F2	0	22.5	22.5	45.0
CK	225	0	0	0

Tahe No.2) was sowed on 14 April 2023 and 15 April 2024 under-membrane drip irrigation. Cotton plant adopted a wide-narrow row arrangement (66 cm + 10 cm), featuring a planting density of 200,000 plants ha^−1^ and a plant spacing of 11.5 cm. The drip irrigation system adopted with an inner diameter of 16 mm, a wall thickness of 0.18 mm, an emitter spacing of 300 mm, and a flow rate of 2.6 L h^−1^. The volume of irrigation water delivered by the drip irrigation system was quantified using a water meter with a measurement accuracy of 0.001 m^3^. In all treatments, the first irrigation was applied via drip within 48 hours after sowing, followed by the second irrigation six days later. After emergence, field management was conducted in accordance with conventional field practices.

300 kg ha^−1^ of urea (46% N), 150 kg ha^−1^ diammonium phosphate (46% P_2_O_5_), and 75 kg ha^−1^ potassium chloride (60% K_2_O) were applied distributed proportionally during each growth stage of cotton. Each block received six irrigation events annually, with 635 m^3^ ha^−1^ per event.

#### 2.2 Soil sampling and analysis

Soil samples were collected using a 5 cm diameter soil auger at 0-20, 20-40, and 40–60 cm before planting and after final harvest of cotton. The sampling time was within 48 hours after each irrigation. At each sampling point, six soil samples were collected and air-dried, passed through a 1 mm sieve, and then used to prepare dilute soil extract solutions with 1:5 of soil water ratio. Soil bulk density and field water capacity were measured using the indoor ring knife methodology. Soil organic matter content was assessed by the dichromate oxidation method. Soil particle composition was determined by using a laser particle size analyzer (Mastersizer 2000, Malvern Instruments Ltd., UK). Soil pH was assessed using pH meter (PHS-2F) (Lu, 2000). Soil salt content was determined by the drying weight method. Soil volumetric water content was determined using oven-drying method.

### 2.3 Plant sampling and analysis

Seedling emergence refers to the point when cotyledons are fully unfolded. Seedling emergence rate was determined when the cotyledonary leaves of over 80% of the cotton seedlings in the field were fully unfolded. For each treatment, 5 sampling points were randomly selected, with 1 m of row length surveyed at each point. The number of sown holes and emerged seedlings within this row length was recorded (Reddy et al., 2017). Cotton root samples were collected as completely as possible using the holistic sampling protocol method (Guo et al., 2024). A rectangular soil volume measuring 40 cm × 10 cm × 60 cm were excavated for root sampling at the end of the cotton seedling stage (63 days after sowing). After collection, soil cubes were placed in individual self-sealing bags and sieved through a 1 mm nylon membrane sieve. Fresh roots were manually separated from soil particles, senescent roots, and other debris, then cleaned, air-dried, and placed in root trays with appropriate water to disperse the roots. Cotton root morphological characteristics were measured by LD-WinRHIZO plant root scanner (Lanende, Inc., CHN).

Root vitality was selected to determined overall root system growth status. Root vitality was measured by the triphenyl tetrazolium chloride method (Clemensson-Lindell, 1994). Ten representative cotton plant samples were selected from each experimental plot at boll opening stage. Cotton plant samples were divided into different organs: roots, stems, leaves, buds, flowers, and bolls. These organs were dried in an oven at 105 °C for 30 minutes and then dried at 80 °C until constant weight to access cotton organ biomass. Root-shoot ratio was calculated as the ratio of root biomass to shoot biomass. Cotton root peroxidase (POD) activity and catalase (CAT) activity were determined via spectrophotometry according to the method described by Zhang and Kirkham (1996). Root superoxide dismutase (SOD) activity was measured by the nitroblue tetrazolium photoreduction method (Giannopolitis and Ries, 1977). Root malondialdehyde (MDA) content was measured by the thiobarbituric acid method (Dhindsa and Matowe, 1981).

During the boll opening stage, three sampling subplots (1 m^2^ for each) were randomly selected in the center of each plot, and cotton bolls were manually collected from each experimental treatment. Subsequently, the weight of the cotton boll and the number of effective bolls per plant were meticulously recorded for determining seed cotton yield.

### 2.4 Calculations

Soil desalination rate (SDR) was used to evaluate the desalination effect of the soil layer and was calculated as follows:


(1) $$\text{SDR}=\frac{S1-S2}{S1}\times 100\%$$

Where S1 is initial soil salt content (g kg^−1^), S2 is soil salt content after end of irrigation (g kg^−1^).

Irrigation water production efficiency (IWPE, kg m^−3^) was determined as follows:


(2) $$\text{IWPE}=\frac{Y}{I}$$

Here, Y indicates seedcotton yield (kg ha^−1^) in each treatment, I is irrigation quota (m^3^ ha^−1^) (Ali and Talukder, 2008).

### 2.5 Statistical analysis

Data analysis was performed using SPSS Statistics 22.0 (IBM, Armonk, USA). One-way analysis of variance (ANOVA) was conducted to detect significant differences among different treatments, followed by Duncan's multiple range test at P < 0.05. The figure was performed with Origin 2024 (Origin Lab, USA). To explore the relationships of soil water and salt dynamics, cotton root characteristics, seedling emergence rate, and yield, correlation analysis was conducted by Origin 2024 using a correlation plot app.

## 3 Results

### 3.1 Soil moisture temporal dynamics

**Figure 1:**
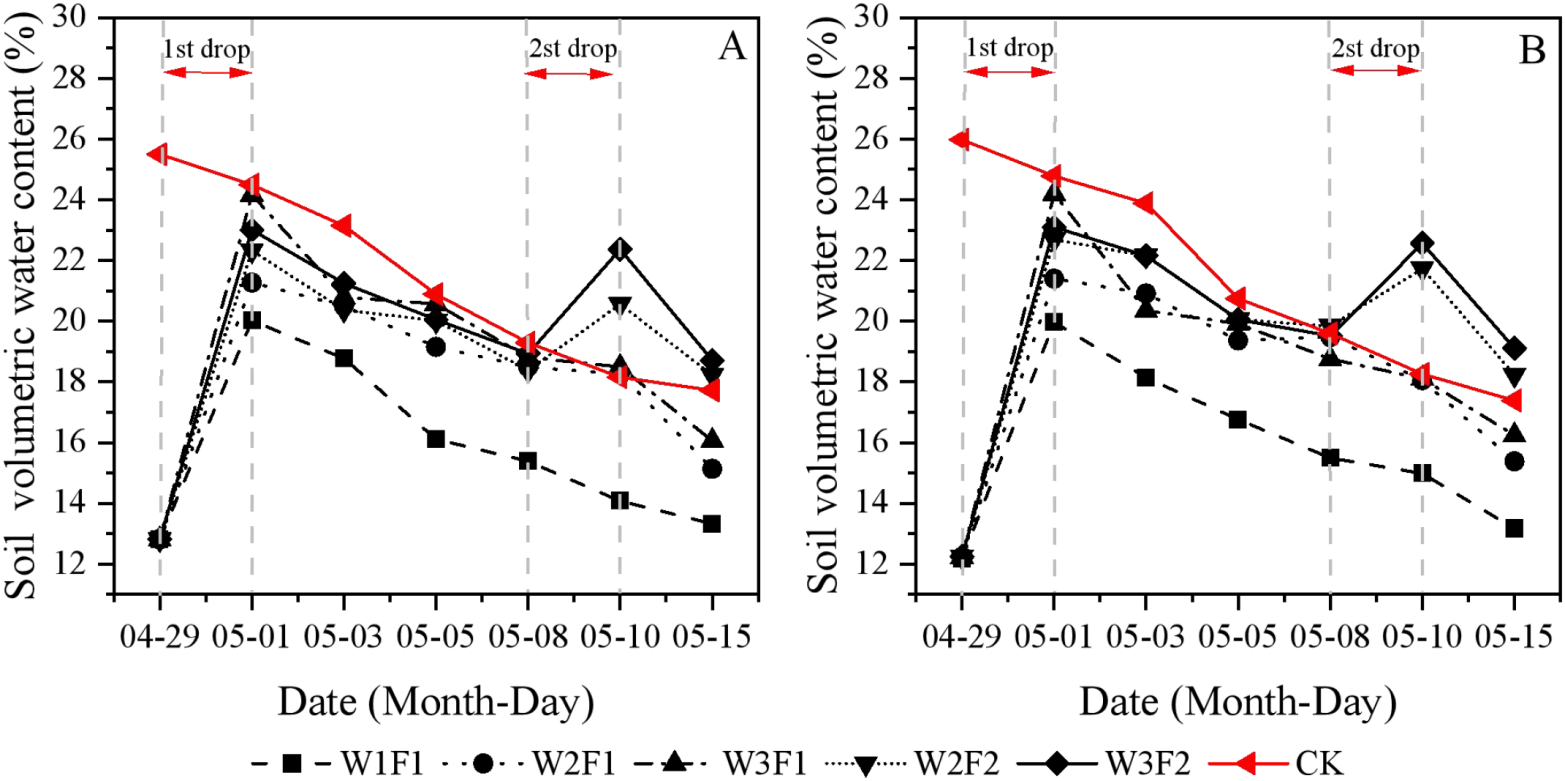
Temporal variations in soil volumetric water content under different emergence water treatments in 2023 **(A)** and 2024 **(B)**. Different lowercase letters indicate significant differences between treatments (p<0.05).

During the seedling period, sharp increases in soil volumetric water content were found immediately after each irrigation, and the trends were similar in the two study years (**Figures 1A, B**). For the one-time irrigation treatment, the peak soil volumetric water content was observed at the emergence stage (April 29th to May 5th). While W3F1 treatment displayed the highest water content among treatments, this value was lower than that of CK. Soil moisture declined gradually as the irrigation period concluded. Soil volumetric water content dynamics in the twice-time irrigation treatment exhibited greater complexity, primarily attributed to the elevated frequency of drip irrigation. Regarding the same irrigation quota, multiple irrigation (F2) resulted in higher soil volumetric water in whole cotton seedling emergence stage compared to single irrigation (F1). Both W2F2 and W3F2 treatments maintained relatively high soil moisture content, which was significantly higher than that of CK during the strong seedling stage (May 8th to May 10th).

### 3.2 Soil salinity temporal and spatial distribution

**Figure 2:**
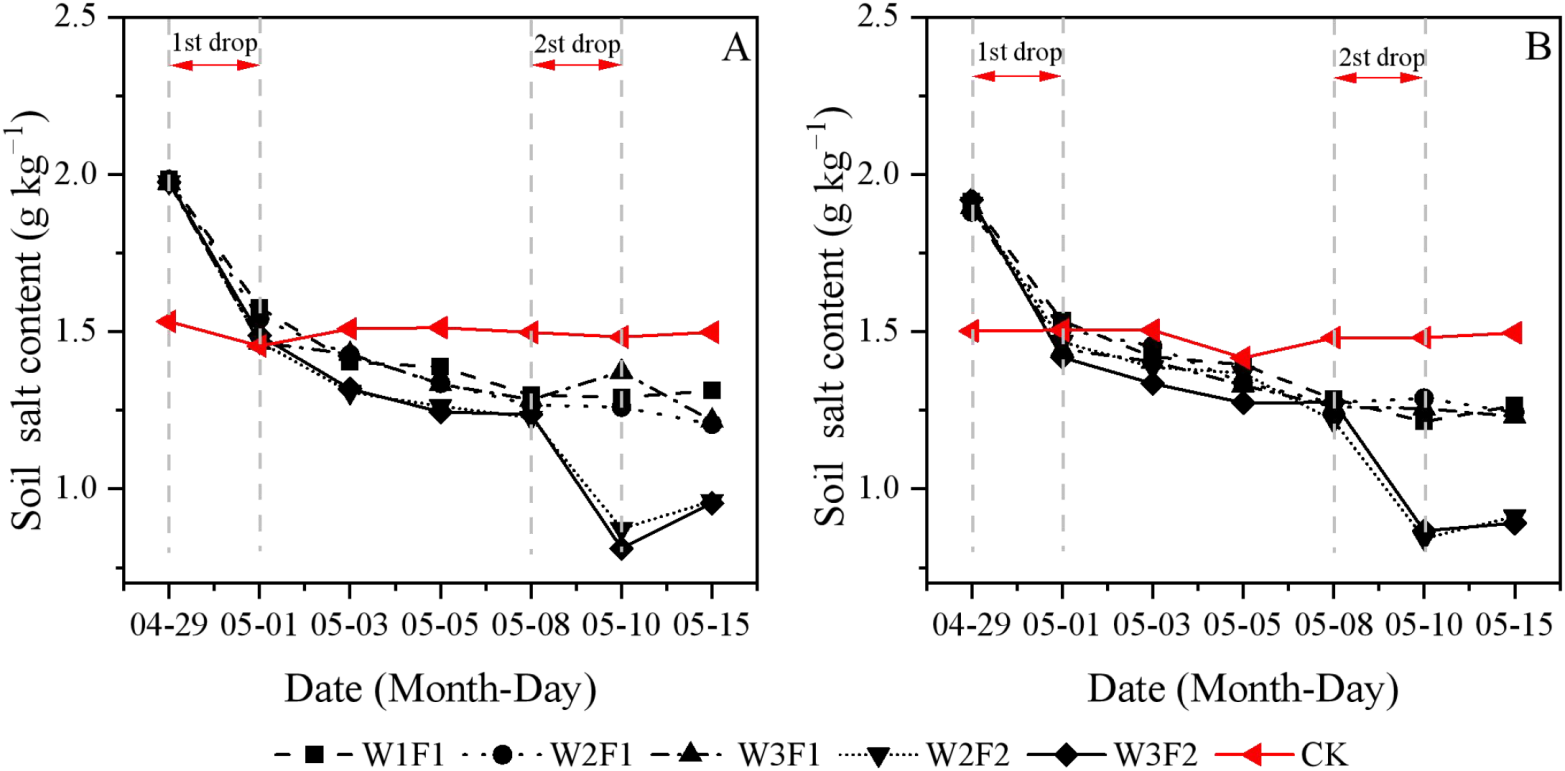
Temporal variations in soil salt content under different emergence water treatments in 2023 **(A)** and 2024 **(B)**. Different lowercase letters indicate significant differences between treatments (p<0.05).

During the seedling period, sharp decreases in soil salt content were observed immediately after each irrigation, and the trends were similar in the two study years (**Figures 2A, B**). For the one-time irrigation treatment, the minimum salt content was showed at the emergence stage (April 29th to May 5th). Soil salt content gradually stabilized as the irrigation period concluded. Soil salt content dynamics in the twice-time irrigation treatment exhibited greater complexity, primarily attributed to the elevated frequency of drip irrigation. Regarding the same irrigation quota, F2 resulted in lower soil salt content in whole cotton seedling emergence stage compared to F1.

**Figure 3:**
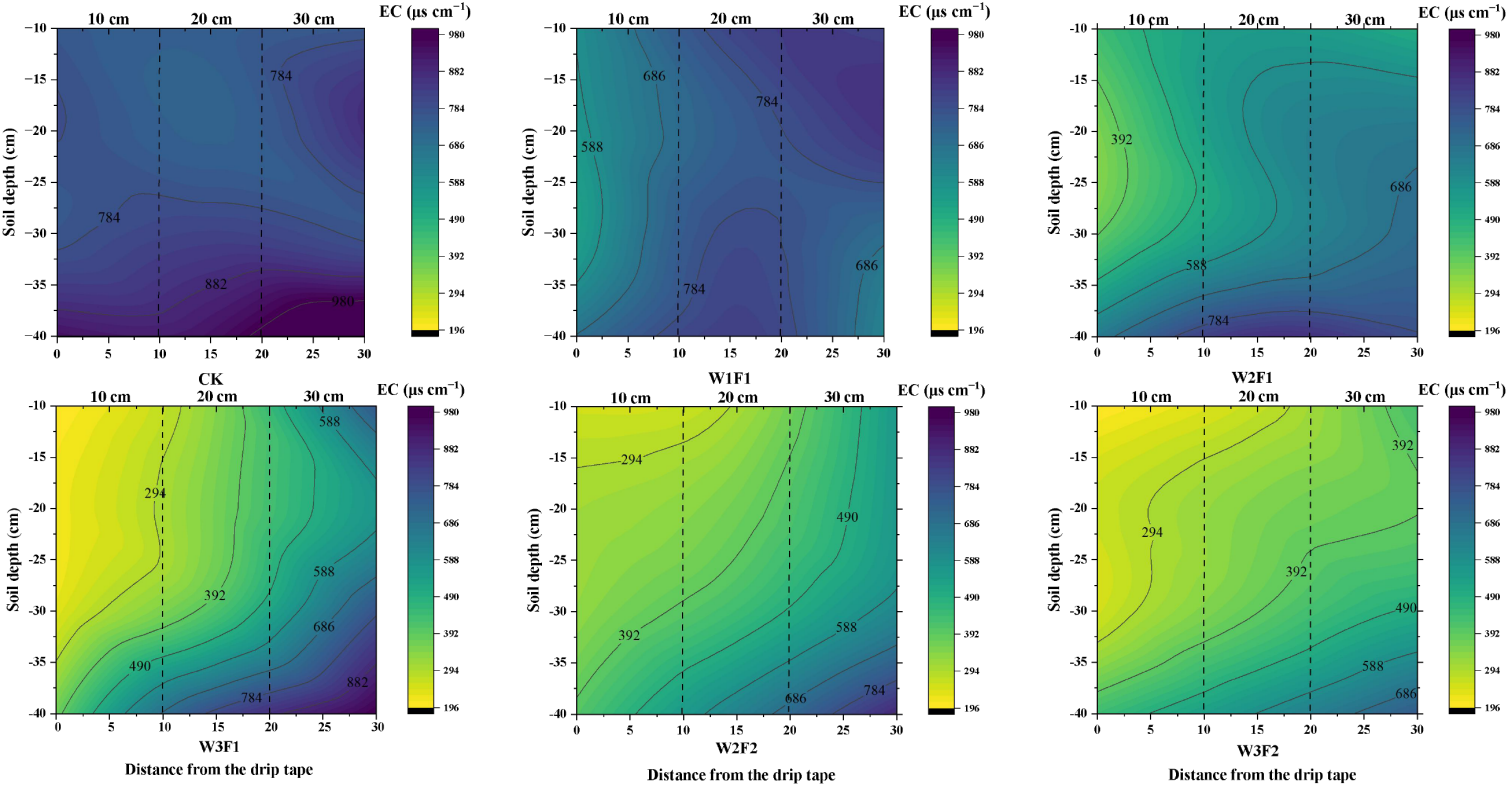
Spatial distribution characteristics of soil electrical conductivity in the soil layer from 0–40 cm averaged over the two years of 2023 and 2024.

Spatial distribution characteristics of soil electrical conductivity (EC) in the soil layer from 0–40 cm averaged over the two years of 2023 and 2024 is depicted in **Figure 3**. The position of the drip tape is set at 0 cm on the horizontal axis. Overall, the soil EC across all treatments was relatively high at a soil depth of approximately 30–40 cm and a horizontal distance of about 30 cm from the drip tape, while it was the lowest near the soil surface and at 0–10 cm from the drip tape. In the horizontal direction, the EC showed an increasing trend with increasing distance from the drip tape, following the order: 10 cm < 20 cm < 30 cm. In the vertical direction, the EC exhibited an irregular decreasing trend as the soil depth increased. Under the same irrigation quota, the EC under F2 was lower than that under F1. As the water application volume increased under constant drip irrigation frequency, a progressive decline was observed in the mean soil EC throughout the 0–40 cm profile depth.

### 3.3 Soil desalination rate

The soil salt content of the CK treatment was significantly lower than that of DSWE treatments before irrigation was applied, and it tended to stabilize during the seedling stage (**Table 3**, Equation 1). Soil desalination rate (SDR) increased significantly and showed notable differences after irrigation. Regarding the same irrigation quota, the soil desalination rate under F2 treatment was significantly higher than that under F1. The two-year average SDR of the W2F2 and W3F2 treatments were 40.2% and 39.5% higher than those of the W2F1 and W3F1 treatments, respectively. For the one-time irrigation treatment, SDR increased as the irrigation amount increased. However, regardless of the number of irrigation, the irrigation amount had no significant effect on the SDR.

**Table 3:** The desalination rate of 0–40 cm soil layer after different emergence water treatments.

Year	Treatment	Initial soil salt content (g kg^−1^)	End of irrigation (g kg^−1^)	Variation	Average desalination rate (%)
2023	W1F1	1.97	1.31 b	0.66	33.8 b
	W2F1		1.21 b	0.76	38.4 b
	W3F1		1.21 b	0.60	38.3 b
	W2F2		0.96 c	1.00	51.1 a
	W3F2		0.96 c	1.00	51.1 a
	CK	1.53	1.49 a	0.04	2.4 c
2024	W1F1	1.95	1.27 b	0.68	35.1 c
	W2F1		1.24 bc	0.71	36.3 bc
	W3F1		1.23 c	0.68	37.4 b
	W2F2		0.91 d	1.04	53.4 a
	W3F2		0.89 d	1.05	54.2 a
	CK	1.51	1.50 a	0.01	0.7 d

Different letters within the same column indicate significant difference at p < 0.05.

### 3.4 Root length density, root vitality and root-shoot ratio

**Figure 4:**
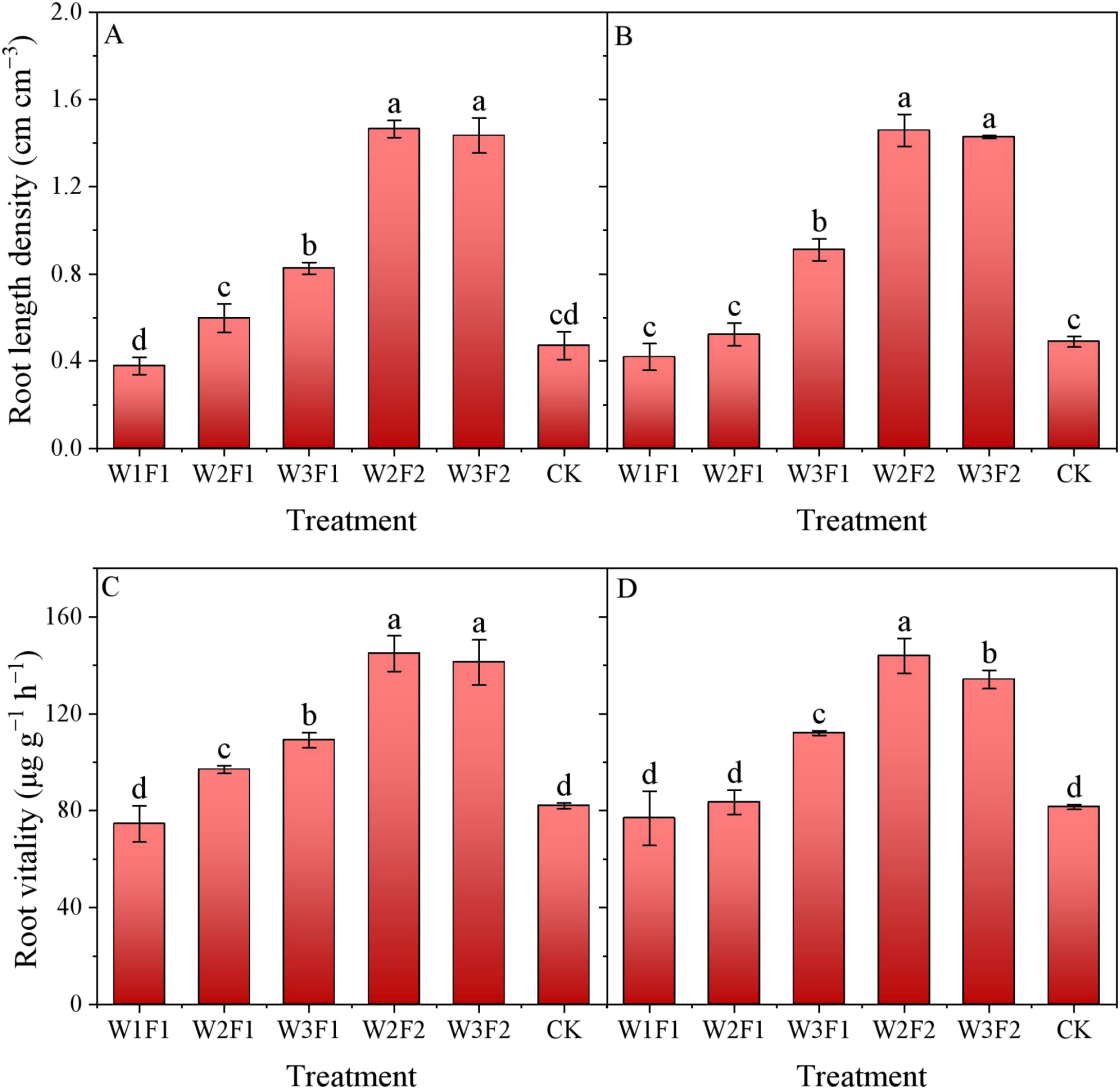
Effect of different emergence water treatments on cotton root length density (RLD) and root vitality in 2023 and 2024. **(A, B)** show the cotton RLD at 30 days after sowing in 2023 and 2024, respectively, and **(C, D)** show the cotton root vitality at 30 days after sowing in 2023 and 2024, respectively. Different lowercase letters indicate significant differences between treatments (p<0.05).

For the same irrigation quota, root length density was consistently greater under F2 treatment than that under F1 (**Figures 4A, B**). The root length density of the W2F2 and W3F2 treatments were 162.5% and 65.3% higher than those of the W2F1 and W3F1 treatments, respectively, across two years. Compared with CK, the root length density under W2F2 and W3F2 treatments increased by 204.4% and 198.2%, respectively, across two years. For the one-time irrigation treatment, the two-year average root length density in W3F1 treatment was significantly higher by 80.6% compared to CK. In contrast, no significant difference was observed among W1F1, W2F1, and CK treatments in 2024. Similarly, there was no significant statistical difference between W2F2 and W3F2 treatments.

The F2 treatment consistently exhibited higher cotton root vitality compared to the F1 under the same irrigation quota (**Figures 4C, D**). The root vitality of the W2F2 and W3F2 treatments were 60.9% and 24.6% higher than those of the W2F1 and W3F1 treatments, respectively, across two years. Compared with CK, the root vitality under W2F2 and W3F2 treatments increased by 76.6% and 68.4%, respectively, across two years. For one-time irrigation treatment, root vitality exhibited an increasing trend as the emergence water increased, and was significantly higher by than that of CK. However, the root vitality under W3F2 treatment was significantly lower than W2F2 treatment in 2024.

**Figure 5:**
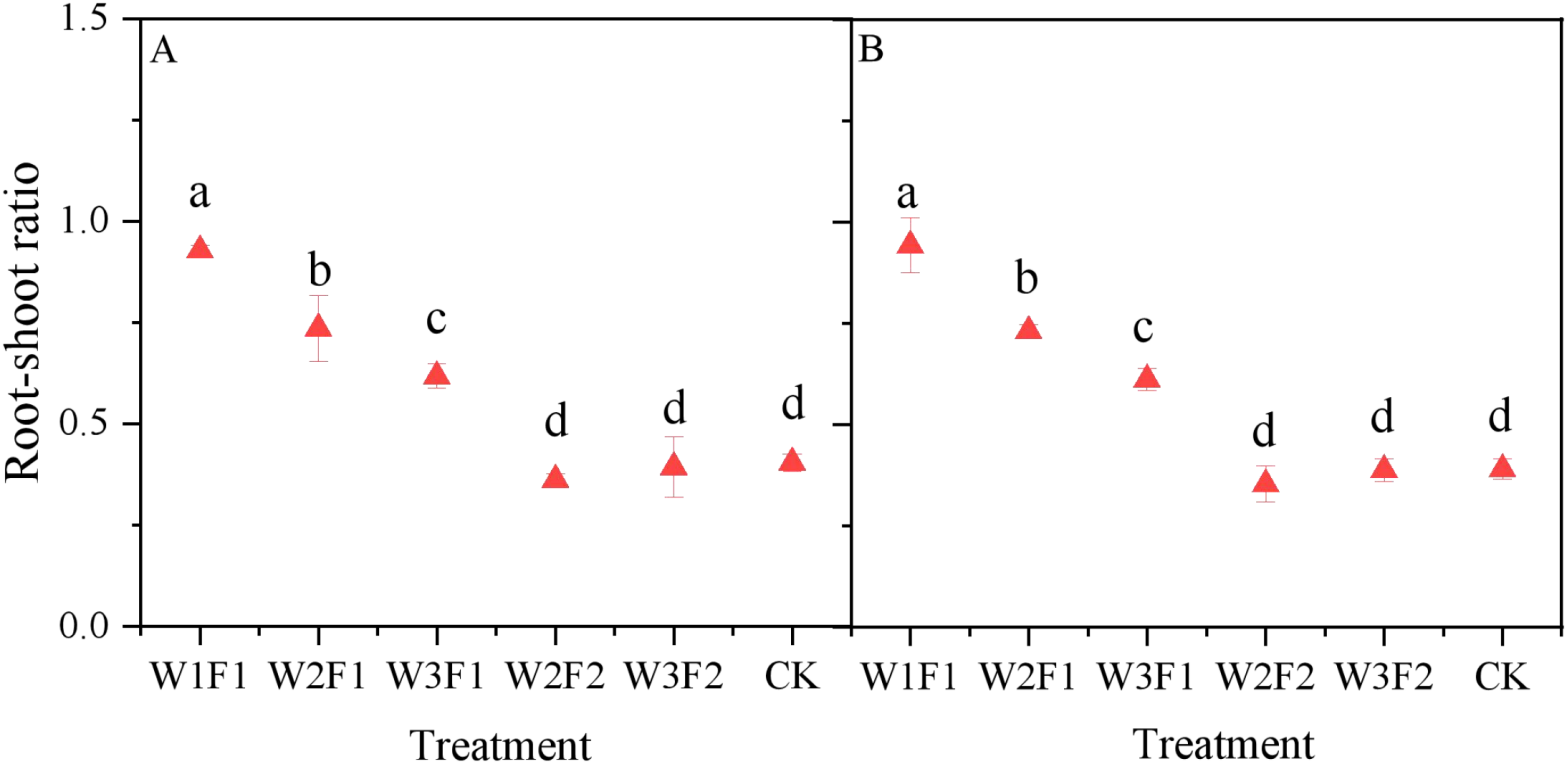
Effect of different emergence water treatments on cotton root-shoot ratio at 30 days after sowing in 2023 **(A)** and 2024 **(B)**. Different lowercase letters indicate significant differences between treatments (p<0.05).

Regarding the same irrigation quota, F2 treatment consistently produced lower cotton root-shoot ratio relative to F1 (**Figures 5A, B**). The root-shoot ratio of the W2F2 and W3F2 treatments were 51.2% and 36.3% lower than those of the W2F1 and W3F1 treatments, respectively, across two years. Compared with CK, the root-shoot ratio under W2F2 and W3F2 treatments decreased by 10.6% and 2.7%, respectively, across two years. For the one-time irrigation treatment, the root-shoot ratio of cotton decreased significantly as the emergence water increased, showing the order W3F1>W2F1>W1F1. However, no significant statistical difference was observed between W2F2 and W3F2 treatments. Moreover, the root-to-shoot ratios in the CK, W2F2, and W3F2 treatments were comparable across the two years.

### 3.5 Root antioxidant enzyme activity

**Figure 6:**
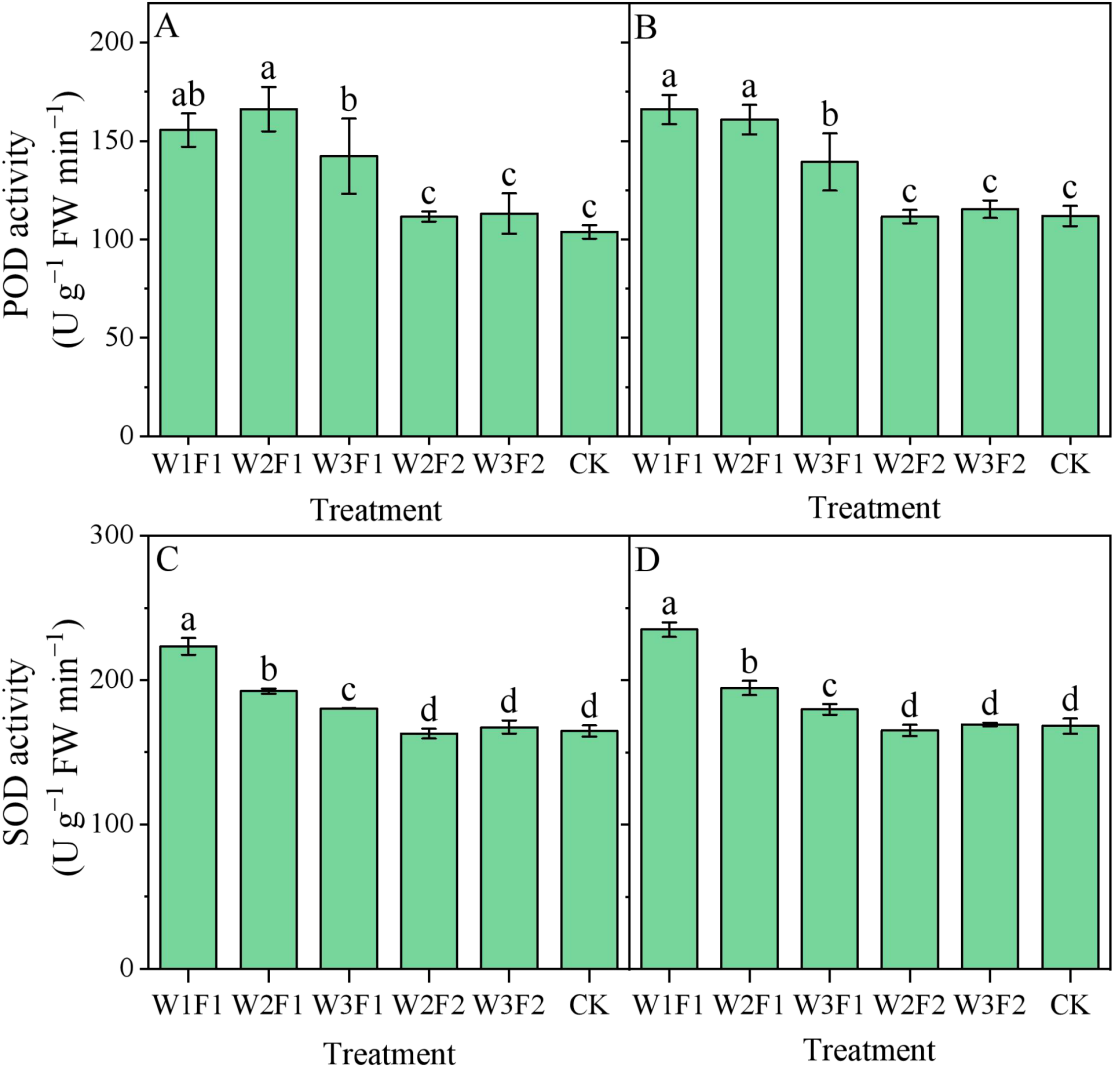
Effect of different emergence water treatments on cotton root peroxidase (POD) activity and superoxide dismutase (SOD) activity at 30 days after sowing in 2023 and 2024. **(A, B)** show the cotton root POD activity at 30 days after sowing in 2023 and 2024, respectively, and **(C, D)** show the cotton root SOD activity at 30 days after sowing in 2023 and 2024, respectively. Different lowercase letters indicate significant differences between treatments (p<0.05).

Regarding the same irrigation quota, F1 treatment had higher cotton root peroxidase (POD) activity relative to F2 (**Figures 6A, B**). The POD activity of cotton root in W2F1 and W3F1 treatments were 23.2% and 46.5% higher than those of the W2F2 and W3F2 treatments, respectively, across two years. For one-time irrigation treatment, POD activity showed a decreasing trend with increasing emergence water. However, there was no significant statistical difference among W2F2, W3F2, and CK treatments.

For one-time irrigation treatment, root superoxide dismutase (SOD) activity showed a decreasing trend with increasing emergence water, with the peak value observed in the W1F1 treatment (**Figures 6C, D**). And there was no significant statistical difference between W1F1 and CK treatments. The SOD activity of cotton root in W2F1 treatment was significantly increased by 18.0% and 17.8% compared to W2F2 treatment, respectively, in 2023 and 2024.

**Figure 7:**
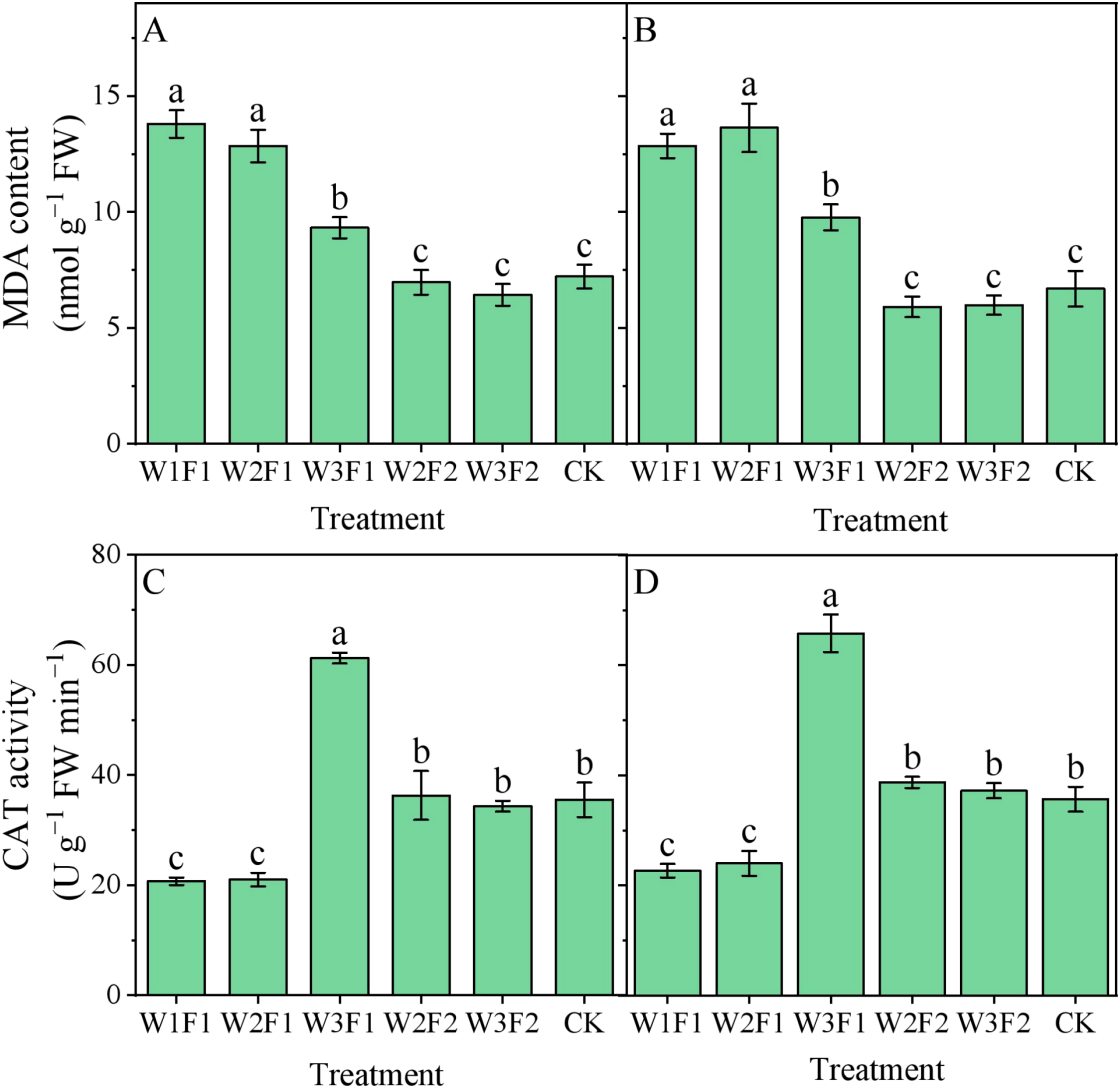
Effect of different emergence water treatments on cotton root malondialdehyde (MDA) content and catalase (CAT) activity at 30 days after sowing in 2023 and 2024. **(A, B)** show the cotton root MDA content at 30 days after sowing in 2023 and 2024, respectively, and **(C, D)** show the cotton root CAT activity at 30 days after sowing in 2023 and 2024, respectively. Different lowercase letters indicate significant differences between treatments (p<0.05).

Regarding the same irrigation quota, F2 treatment had lower cotton root malondialdehyde (MDA) content relative to F1 (**Figures 7A, B**). The MDA content of cotton root in W2F2 and W3F2 treatments were 51.2% and 34.9% lower than those of the W2F1 and W3F1 treatments, respectively, across two years. For one-time irrigation treatment, MDA content showed a decreasing trend with increasing emergence water, with the peak value observed in the W1F1 treatment. Compared with CK, the MDA content under W1F1 treatment increased by 92.2% and 91.3%, respectively, in 2023 and 2024. However, there was no significant statistical difference among W2F2, W3F2, and CK treatments.

It was observed that root catalase (CAT) activity was highest under the W3F1 treatment (**Figures 7C, D**), being significantly increases of 78.5% and 77.6% compared to CK and W3F2 treatment, respectively, across two years. For the one-time irrigation treatment, the CAT activity of cotton root in W2F1 and W1F1 treatments were significantly decreased by 65.9% and 65.6% compared to W3F2 treatment. However, there was no significant statistical difference among W2F2, W3F2, and CK treatments.

### 3.6 Cotton seedling emergence rate, yield, and IWPE

**Figure 8:**
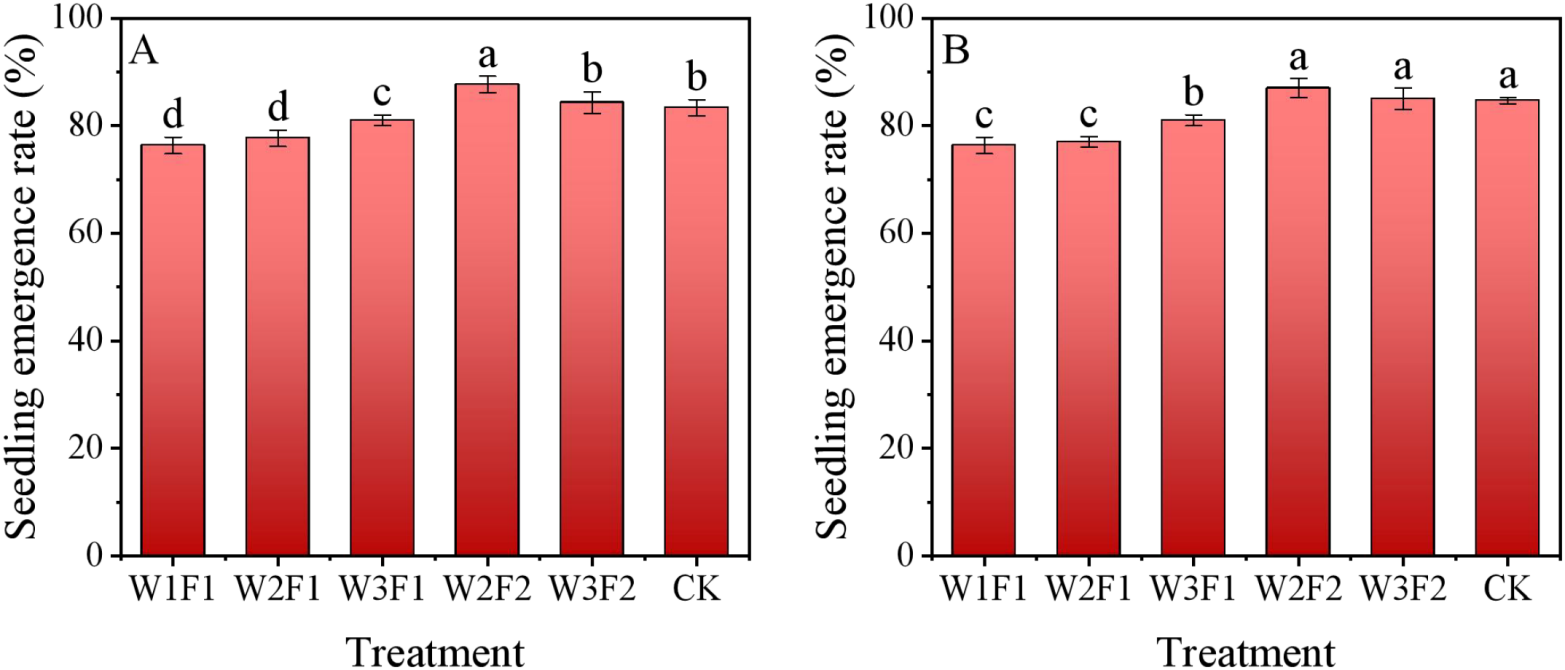
Cotton seedling emergence rate under different emergence water treatments in 2023 and 2024. Different letters represent significant difference among treatments at p< 0.05.

Regarding the same irrigation quota, F2 treatment consistently produced higher cotton seedling emergence rate relative to F1 (**Figures 8A, B**). For the one-time irrigation treatment, cotton seedling emergence rate under W3F1 treatment was significantly higher than W1F1 and W2F1, whereas no significant difference was found between W1F1 and W2F1 treatments. W2F2 treatment exhibited maximal seedling emergence rate, but no statistical difference between CK and W2F2 treatments in 2024.

Different emergence water treatments significantly affected both the number of bolls per plant and seedcotton yield, but had no significant effect on boll weight (**Table 4**). For the one-time irrigation treatment, boll numbers and seed cotton yield significantly increased as the irrigation amount increased. However, the irrigation amount had no significant effect on the number of bolls per plant and seedcotton yield under treatments with two-time irrigation. Regarding the same irrigation quota, F2 treatment resulted in higher the number of bolls per plant and seedcotton yield compared to F1. Compared with W2F1 treatment, the number of bolls per plant was increased by 22.8% and 23.8% under W2F2 treatment in 2023 and 2024, respectively. W2F2 treatment had the highest seedcotton yield, which were significantly increased by 22.5% and 24.5% compared to W2F1 treatment in 2023 and 2024, respectively. However, there was no significant difference in boll numbers and seedcotton yield between CK, W2F2, and W3F2 treatments. Furthermore, IWPE was higher in all DSWE treatments than that of CK (Equation 2). Similarly, W2F2 treatment exhibited the highest IWPE, which were significantly increased by 58.3% and 55.8% compared to CK in 2023 and 2024, respectively. Compared with W3F2 treatment, the IWPE under W2F2 treatment increased by 3.4% and 4.0%, respectively, in 2023 and 2024.

**Table 4:** Cotton yield and water production efficiency under different water treatments.

Year	Treatment	Irrigation quota (m^3^ ha^−1^)	Boll numbers per plant (No)	Boll weight (g)	Seedcotton Yield (kg ha^−1^)	IWPE (kg m^−3^)
2023	W1F1	3275	5.2 d	4.38 a	4520.9 d	1.38 d
	W2F1	3425	5.5 c	4.37 a	4736.80 c	1.38 d
	W3F1	3500	6.1 b	4.39 a	5385.9 b	1.54 c
	W2F2	3425	6.8 a	4.33 a	5823.7 a	1.71 a
	W3F2	3500	6.6 a	4.38 a	5752.8 a	1.64 b
	CK	5300	6.7 a	4.27 a	5721.2 a	1.08 e
2024	W1F1	3275	5.2 d	4.36 a	4580.6 d	1.40 d
	W2F1	3425	5.5 c	4.41 a	4776.0 c	1.39 d
	W3F1	3500	6.2 b	4.39 a	5418.2 b	1.55 c
	W2F2	3425	6.7 a	4.43 a	5851.5 a	1.71 a
	W3F2	3500	6.6 a	4.38 a	5747.6 a	1.64 b
	CK	5300	6.7 a	4.36 a	5810.8 a	1.10 e

IWPE, Irrigation water production efficiency. Data are showed in mean value (n=3). Different letters within the same column indicate significant difference at p < 0.05.

### 3.7 Correlation analysis

**Figure 9:**
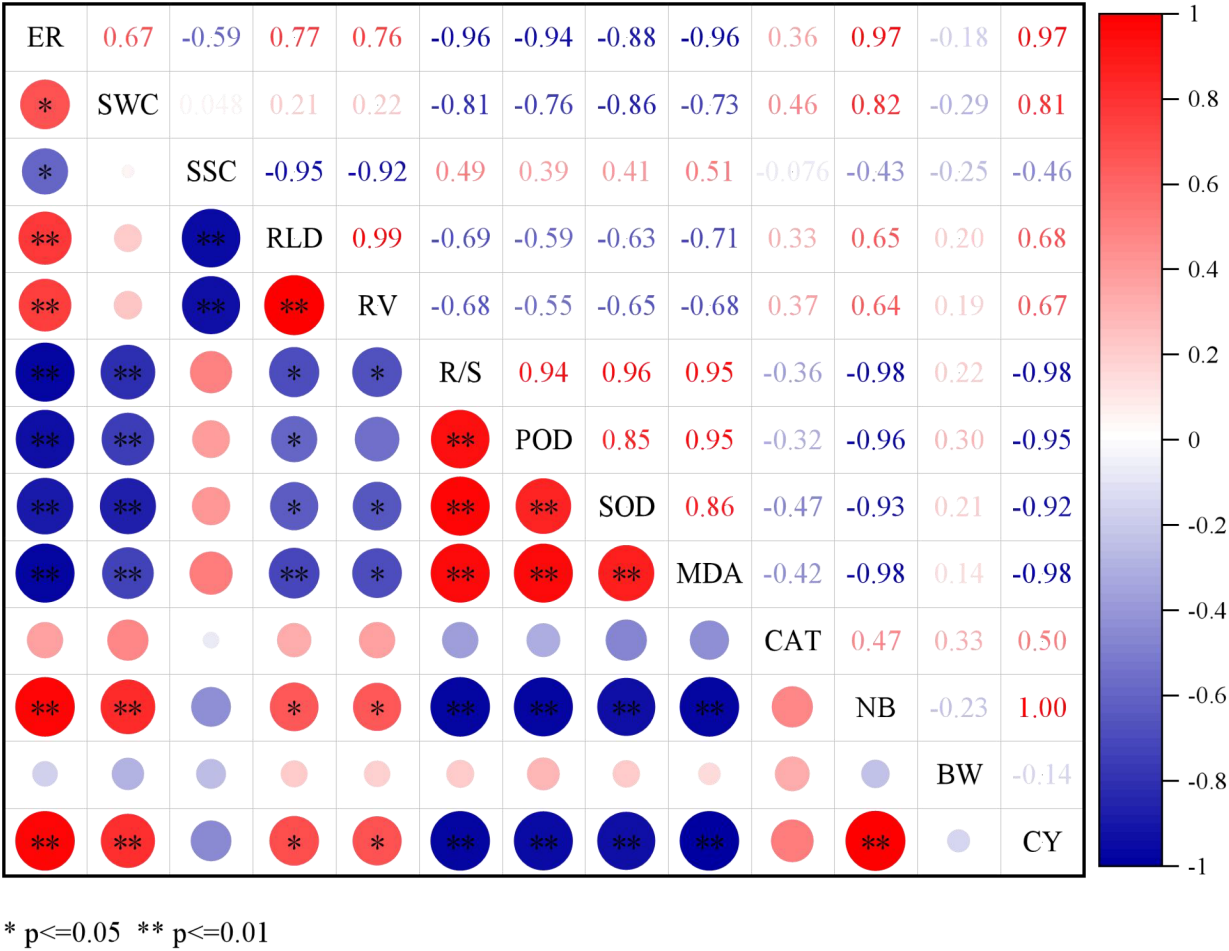
Heatmap demonstrating correlation relationship among all variables examined. Correlation coefficients vary from 1.0 (red) to −1.0 (blue). * and ** represent significant differences at p < 0.05 and p < 0.01 probability level. ER, emergence rate; SWC, soil volumetric water content; SSC, soil salt content; RLD, root length density; RV, root vitality; R/S, root-shoot ratio; POD, root peroxidase activity; SOD, root superoxide dismutase activity; MDA, root malondialdehyde content; CAT, catalase activity; BN, boll number; BW, boll weight; CY, seedcotton yield.

Correlation analysis (**Figure 9**) showed that soil volumetric water content, root vitality and RLD were positively correlated with cotton seedling emergence rate. The soil salt content was negatively correlated with cotton seedling emergence rate, RLD and root vitality. The soil salt content was negatively correlated with cotton seedling emergence rate, root length density and root vitality. The seedcotton yield showed significant positive correlations with emergence rate, soil volumetric water content, root length density, root vitality, and boll number, while having significant negative correlations with root-shoot ratio, root POD, SOD, and MDA content. Relationships of soil water and salt distribution, SDR, cotton root distribution, seedling emergence rate, IWPE, and seedcotton yield.

## 4 Discussion

### 4.1 Influence of DSWE water regulation on soil water and salt dynamics, desalination efficiency

Soil moisture content is a critical environmental factor that influences cotton emergence and growth (Hou et al., 2022b). Insufficient soil moisture fails to meet the normal water requirements of seedling establishment, thereby hindering cotton development. Conversely, excessive soil moisture can reduce soil temperature, which negatively affects cotton emergence (Hu et al., 2009). The dry sowing and wet emergence (DSWE) water regulation method exerts distinct effects on soil water and salt dynamics, owing to its unique sequence of dry seeding followed by targeted irrigation to facilitate seedling emergence. In this study, W2F2 and W3F2 treatments maintained relatively high soil moisture content, which was significantly higher than that of CK, owing to the increased frequency of drip irrigation during the strong seedling stage (**Figures 1A, B**). Controlled drip irrigation frequencies can maintain the stability of soil moisture in the sowing layer (Chilundo et al., 2018; He et al., 2023), reduce drastic fluctuations in moisture, and provide a continuously moist microenvironment for seed germination. This is particularly true under conditions of high temperature and strong evaporation, where a high irrigation frequency can offset evaporation losses (Nabi and Mullins, 2008). Furthermore, by avoiding pre-sowing flooding or excessive irrigation, controlled drip irrigation amount and frequencies effectively reduces initial soil saturation. Subsequent controlled wetting facilitates the maintenance of optimal root zone moisture levels over extended periods, compared to over-irrigation practices. This is primarily due to the slower evaporation rate from drier surface layers in comparison to that from fully saturated soil.

High soil salt content will affect seed germination and slow down cotton seedling growth, while a suitable water-salt environment can promote cotton growth (Heng et al., 2022). This study found that soil salt content showed a gradual reduction with increased water volume at emergence and higher drip irrigation frequency (**Figures 2A, B**). This is consistent with the findings of Wang and Wang (2024), who reported that optimized drip irrigation frequencies have demonstrated effectiveness in reducing excessive soil salt accumulation and promoting the downward movement of salts away from the root zone. Controlled drip irrigation amount and frequencies can continuously keep the soil surface moist and reduce intense evaporation-evaporation being the main driver of salt return-thereby inhibiting the accumulation of salts in the surface layer (Ren et al., 2021). The soil EC was the lowest near the soil surface (the shallow root distribution zone) and at 0–10 cm from the drip tape (**Figure 3**). This demonstrates that drip irrigation water can precisely act on the key zones with dense crop roots, achieving root zone desalination through wetting and leaching, thereby creating a low-salt environment for crop germination, water uptake, and nutrient absorption. In contrast, the EC was relatively higher at a soil depth of 30–40 cm (the bottom of the root zone) and 30 cm from the drip tape (the edge of the wetted zone) (**Figure 3**). Therefore, drip irrigation can precisely migrate salt in the root zone to non-critical areas by optimizing irrigation frequency and volume, realizing root zone desalination (Chauhdary et al., 2020). Meanwhile, frequent irrigation can continuously and slowly leach salts from the sowing layer, maintaining a low-salt environment. This is also confirmed by our results on SDR (**Table 3**). For the CK treatment, due to the larger irrigation volume, the salt leaching effect was better in the early stage, resulting in a lower salt content at the early sowing stage. Among the various DSWE water regulation treatments, W2F2 and W3F2 treatments with more irrigation events and larger irrigation volumes, achieved better salt leaching in the soil surface layer and thus higher SDR (**Table 3**).

The DSWE technique achieves the goal of regulating salt with water and promoting seedling growth with water by controlling irrigation volume and frequency, appropriate irrigation volume and frequency can maintain sufficient and stable soil moisture in the sowing layer. Meanwhile, through moderate salt leaching and evaporation inhibition, the salt concentration in the surface layer is reduced, creating an environment with "adequate water, low salt, and suitable aeration" for seed germination.

### 4.2 Influence of DSWE water regulation on cotton root growth

The status of soil moisture and salt is closely related to the growth and development of plant roots (Chen et al., 2020, Chen et al., 2018). In our study, root length density (RLD) and root vitality were increased with both the amount of seedling water and the frequency of drip irrigation (**Figures 4A–D**). A reasonable amount and frequency of seedling emergence water can keep the root system in a "moist but not waterlogged" environment, ensuring normal respiration, sufficient energy supply, and high root vitality, which is beneficial to nutrient absorption and seedling growth (Wang et al., 2020). When the depth of the soil moist layer is appropriate, the root system can access water without excessive elongation. Meanwhile, the low-salt environment reduces osmotic inhibition, promoting coordinated growth of the taproot and lateral roots and resulting in a relatively high RLD. Insufficient irrigation amount or frequency leads to drought in the soil surface layer, causing salt to easily concentrate in the root zone, which in turn results in osmotic stress and ion toxicity, and a decrease in root vitality.

Root antioxidant enzymes, including superoxide dismutase (SOD), peroxidase (POD), and catalase (CAT), act as key protective mechanisms against abiotic stresses such as salt and drought stress (Noreen and Ashraf, 2009). Changes in their activities reflect the intensity of the root system's stress response, when soil salts are effectively leached and soil moisture is stable, the root system is subjected to low-level stress, and antioxidant enzyme activities remain at a basal level (to maintain oxidative balance for normal metabolism) without excessive activation, thus avoiding energy waste. However, drought combined with high salt levels induces oxidative stress, prompting root cells to generate large amounts of reactive oxygen species (ROS). In response, the antioxidant enzyme system is significantly activated, with increased activities of SOD, POD, and CAT to scavenge ROS and protect cell membrane structures. In this study, an increase in amount and frequency of seedling emergence water effectively reduced the POD (**Figures 6A, B**), SOD (**Figures 6C, D**), catalase CAT activity (**Figures 7C, D**) and MDA (**Figures 7A, B**) content of the cotton root. The possible reason is that the elevated amount and frequency of seedling emergence water can effectively control salt in the root zone, reduce salt stress, and avoid oxygen deficiency (Hu et al., 2025). Consequently, antioxidant enzyme activities remain at a relatively low level, indicating that the root system is in a superior physiological state where more energy is allocated to growth rather than stress response. This is also confirmed by our results on the root-shoot ratio (**Figures 5A, B**), which appropriate water management treatments resulted in a lower cotton root-shoot ratio. Overall, in the DSWE technique, a combination of moderate irrigation amount (meeting the water demand at the seedling stage without waterlogging) and reasonable frequency (applied multiple times to maintain a moist, low-salt surface layer) constitutes an optimal approach. This combination not only ensures that root vitality and RLD remain at relatively high levels, promoting nutrient uptake and root expansion, but also reduces salt or drought stress, keeping antioxidant enzyme activities within a moderate range and avoiding excessive energy consumption. Ultimately, it facilitates the robust development of cotton root systems at the seedling stage, laying a solid foundation for subsequent growth.

### 4.3 Influence of DSWE water regulation on cotton seedling emergence rate, yield, and irrigation water production efficiency

The intricate interplay between soil-root environmental factors and root growth plays a pivotal role in optimizing crop establishment and enhancing agricultural productivity within arid agroecosystems (Calleja-Cabrera et al., 2020), especially in the desert-oasis ecotones of Xinjiang. The DSWE water regulation method exerts significant and interconnected impacts on cotton seedling emergence rate, final yield, and IWPE. These effects be attributed to optimize early soil conditions, balance water use, and promote root growth. In this study, cotton seedling emergence rate were higher in W2F2 treatment than other treatments (**Figures 8A, B**). This may be because the irrigation management practice of fewer and more frequent irrigation, through sowing in dry soil followed by targeted irrigation (focused on the seed zone), enables DSWE to ensure that seeds receive sufficient moisture to promote germination, while avoiding excessive water that could cause soil crusting or seed rot. In this way, uneven emergence caused by drought stress or waterlogging can be prevented. This is also proved by our results on the root antioxidant enzymes activity (**Figures 6**, **7**), which appropriate water management treatments ensures a favorable environment for cotton to grow in the soil’s root zone. The positive effects on emergence result in yield benefits, which are further reinforced by advantages observed in subsequent growth stages (Dai et al, 2024). A higher and more uniform emergence rate ensures a consistent plant population, reducing gaps in the field and maximizing light interception, nutrient uptake, and resource utilization during the growing season. This avoids yield losses from sparse or uneven stands. DSWE treatments promotes healthier root development (as discussed in 4.2) and vigorous seedling growth by minimizing stress. Robust seedlings better withstand later abiotic stresses (e.g., drought, salinity) and pests, maintaining higher photosynthetic capacity through flowering and boll formation (Ding et al., 2024). Our data indicated that both cotton boll numbers per plant and seed cotton yield increased gradually as emergence watering was increased (**Table 4**). Notably, the high-frequency irrigation treatment resulted in significantly higher values for both parameters. Fewer and more frequent irrigation practice through the regulation of soil moisture and the reduction of salt accumulation within the root zone across the growth cycle, particularly when integrated with appropriate post-emergence irrigation, to promote sustained nutrient uptake and improved boll retention. In saline environments, this mechanism effectively prevents salt stress-induced premature leaf senescence and boll abortion (Pettigrew, 2004). Over the two years, the highest irrigation water production efficiency (IWPE) was obtained from the fewer and more frequent irrigation practice (W2F2) (**Table 4**). Unlike diffuse irrigation or pre-sowing heavy irrigation, DSWE uses fewer and more frequent irrigation focused only on the seed zone, minimizing water loss through deep percolation or evaporation from non-critical soil layers. This lowers the total early-season irrigation volume, while deep roots stimulated by controlled early moisture access water from lower soil layers, thereby reducing the need for frequent irrigation and enhancing overall water productivity (Lin et al., 2024; Xiao et al., 2023). Therefore, the W2F2 treatments of DSWE irrigation scheme is recommended as a sustainable production strategy for cotton fields in the arid southern region of Xinjiang, China. It not only ensures seedling emergence, promotes root growth, and drives yield improvement by increasing the number of cotton bolls, but also enhances water use efficiency and reduces agricultural irrigation water consumption (Wang et al., 2024). This study revealed the effects of different DSWE water regulation treatments on the soil water-salt environment (water content, salt content, and SDR), cotton root growth (RLD and root-shoot ratio), and root physiological status (root vitality, antioxidant enzyme activity, and MDA content). It clarified that optimizing the water-salt environment and alleviating root oxidative stress can promote root development, thereby enhancing yield and irrigation efficiency, which provides mechanistic support for the precise regulation of cotton irrigation in arid and semi-arid regions.

## 5 Conclusion

Under the same irrigation quota, multiple irrigations (F2) maintained higher soil water content and lower salt content compared to single irrigation (F1). Specifically, the W2F2 and W3F2 treatments exhibited significantly higher soil moisture levels and SDR than the control (CK), with average SDR of W2F2 and W3F2 treatments being 40.2% and 39.5% higher, respectively, than their corresponding F1 treatments. Multiple irrigations (F2) significantly enhanced RLD and root vitality. W2F2 showed a 162.5% increase in RLD and a 60.9% increase in root vitality compared to W2F1 treatment, while simultaneously reducing the root-shoot ratio. In contrast, single irrigation (F1) resulted in elevated activities of root POD, SOD, and CAT activities, as well as increased MDA content, suggesting that it may induce more severe oxidative stress in roots. The W2F2 treatment achieved a seed cotton yield that was 23.5% higher than that of W2F1 treatment, and it also demonstrated the highest IWPE, which was 57.05% higher than that of CK. In conclusion, fewer and more frequent irrigations (W2F2 treatment) can enhance cotton emergence rate, yield, and irrigation efficiency by optimizing the soil water-salt environment and promoting root growth and physiological status, thereby representing a more effective irrigation management strategy. Given the inherent errors of traditional sampling methods and the rise of smart agriculture, future research should be deeply integrated with smart agriculture to establish an integrated soil-water-crop-meteorology monitoring and regulation system, and reducing reliance on manual work.

## Data Availability

The original contributions presented in the study are included in the article/supplementary material. Further inquiries can be directed to the corresponding authors.
